# Assessment of causal associations between uric acid and 25-hydroxyvitamin D levels

**DOI:** 10.3389/fendo.2022.1024675

**Published:** 2022-12-13

**Authors:** Yingdong Han, Yun Zhang, Xuejun Zeng

**Affiliations:** ^1^ Department of family medicine, Peking Union Medical College Hospital, Chinese Academy of Medical Sciences, State Key Laboratory of Complex Severe and Rare Diseases (Peking Union Medical College Hospital), Beijing, China; ^2^ Division of General Internal Medicine, Department of medicine, Peking Union Medical College Hospital, Chinese Academy of Medical Sciences, State Key Laboratory of Complex Severe and Rare Diseases (Peking Union Medical College Hospital), Beijing, China

**Keywords:** 25-hydroxyvitamin D, mendelian randomization, causality, inverse-variance weighting method, uric acid

## Abstract

**Background:**

Previous observational studies have revealed the association between serum uric acid and 25-hydroxyvitamin D. However, the causality and the direction of the associations remain unknown. Thus, we performed a two-sample bidirectional Mendelian Randomization (MR) analysis to investigate the causal association between uric acid and 25-hydroxyvitamin D and to determine the direction of the association.

**Method:**

Based on the summary-level GWAS data from large genome-wide association studies, several steps were taken in our analysis to select eligible single-nucleotide polymorphisms (SNPs), which were strongly related to exposure as the instrumental variables. We used different analytical methods, such as inverse-variance weighting (IVW) method, weighted median, MR-Egger regression, and weighted mode method, to make our result more robust and reliable. The IVW method was used as the primary analysis. The Cochran’s Q test, MR-Egger intercept test, MR-PRESSO method, and “leave-one-out” sensitivity analysis was performed to evaluate the heterogeneities, horizontal pleiotropy, and robustness of the results. MR analyses were also conducted using genetic risk scores (GRS) as instrumental variables in both directions by using the same summary-level GWAS data.

**Results:**

Our two-sample MR analysis suggested a causal association of genetically predicted uric acid on 25-hydroxyvitamin D [IVW method: β(SE), −0.0352(0.0149); *p* = 0.0178], which suggested that a per mg/dl increase in uric acid was associated with a decrease of 0.74 nmol/L of 25-hydroxyvitamin D, and the above results remained stable in the sensitivity analysis. By contrast, four MR methods suggested no causal relationship of 25-hydroxyvitamin D on serum uric acid [IVW β(SE), 0.0139 (0.0635); *p* = 0.826; MR-Egger β(SE), 0.0671 (0.108); *p* = 0.537; weighted median β(SE), 0.0933 (0.0495); *p* = 0.0598; weighted mode β(SE), 0.0562 (0.0463); *p* = 0.228, respectively]. After excluding the SNPs, which were associated with confounding factors and outlier SNPs, the IVW method suggested that there was still no causal association of 25-hydroxyvitamin D on serum uric acid. The GRS approach showed similar results.

**Conclusions:**

Serum uric acid may causally affect the 25-hydroxyvitamin D levels, whereas the causal role of 25-hydroxyvitamin D on uric acid was not supported in our MR analysis. Our findings suggest that increased levels of uric acid should prompt investigation for vitamin D deficiency.

## 1 Introduction

In the past decades, serum uric acid (SUA) levels had increased significantly due to changes in dietary patterns and improvement of living conditions, such as increased consumption of fructose-containing foods, seafood, and organ meat. An epidemiological study from the US suggested that the overall prevalence of hyperuricemia increased from 18.2% to 21.4%, and the prevalence of gout increased from 2.7% to 3.9% (1). Another epidemiological study found that the overall prevalence of hyperuricemia among Chinese increased from 11.0% to 14.0% during 2015–2016 and 2018–2019 (2). SUA has a protective effect in neurodegenerative diseases, such as dementia and Parkinson’s disease, because extracellular urate is an antioxidant with potentially anti-inflammatory effects (3). However, hyperuricemia brings common health problems and affects nearly 21% of the US adults. Occult deposition of monosodium urate may induce inflammation, mechanical damage of the joint, and even systemic consequences. Hyperuricemia is the precursor of gout, the joint and kidney being the mostly involved organs. In addition, hyperuricemia and gout are usually accompanied by various comorbidities, such as hypertension, diabetes, metabolic syndrome, and dyslipidemia (4–7).

Osteoporosis is a common but often overlooked systemic disease by doctors, which is characterized by the microarchitectural deterioration of bone tissue, low bone mass, bone fragility, and increased risk of fractures (8, 9). Around 10 million Americans over the age of 50 have osteoporosis, and approximately 1.5 million suffer fragility fractures each year. The economic and mortality burden attributed to fragility fracture is significant: annually, approximately 31,000 deaths occur within 6 months of hip fracture in the US, costing about $17.9 billion (10).

Several studies found that SUA plays an important role in the development of osteoporosis (11). SUA have been significantly associated with higher risk of hip fracture in men (12). Monosodium urate crystals promote the development of osteoclasts within tophi and their vicinity and reduce the activity of osteoblasts (13). Hyperuricemia could suppress the expression and activity of 1-α hydroxylase and has been associated with vitamin D deficiency (14). Vitamin D has an established role in regulating the normal homeostasis of calcium and phosphorus, which regulates the activity of osteoblasts and osteoclasts, promotes bone formation, and prevents osteoporosis (15).

Vitamin D deficiency and insufficiency is a global health problem affecting over one billion people worldwide. Sunlight exposure remains the major source of vitamin D for most people, and insufficient exposure to sunlight is the primary cause of vitamin D deficiency among all ages (16). Vitamin D from both sun exposure and exogenous intake from diet is metabolized in the liver to 25-hydroxyvitamin D [25(OH)D], which is the most widely used marker and estimator of vitamin D status. Observational studies show that vitamin D deficiency, aside from maintaining the homeostasis of calcium and phosphorus, is linked to a variety of clinical conditions, such as cardiovascular disease, type 2 diabetes mellitus, and autoimmune disease (17–19).

Previous studies on the association between SUA and 25(OH)D have not reached an agreement. Seibel’s study suggests a positive correlation between SUA and 25(OH)D in multiple regression analyses (20). Our previous cross-sectional study revealed that 25(OH)D were inversely associated with hyperuricemia in the general adult population in the US (21). Vitamin D deficiency can cause secondary hyperparathyroidism and lead to an increase in the serum parathyroid hormone (PTH) concentration (22). PTH can affect the secretion and transport of uric acid and lead to hyperuricemia (23, 24). Hypovitaminosis D is associated with insulin resistance (25), while insulin resistance is inversely correlated to the renal clearance of SUA and can lead to hyperuricemia (26). In addition, SUA suppresses 1-α hydroxylase protein and mRNA expression and thereby reduces the concentrations of 1,25(OH)_2_D (14).

The above controversial results suggested that confounding and reverse causation can create spurious associations in observational studies. There is no previous study that explored the causal relationship between SUA and 25(OH)D. The limitations of previous observational studies can be effectively addressed by using Mendelian Randomization (MR) analysis. We conducted this study using summary-level GWAS data to explore the causal association of SUA and 25(OH)D with two-sample bidirectional MR analysis

## 2 Materials and methods

**Figure 1:**
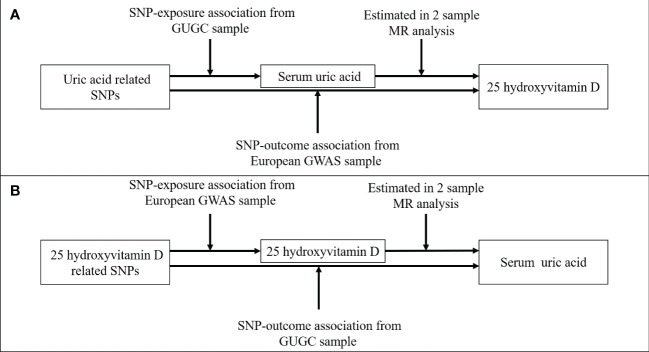
Two-sample bidirectional Mendelian Randomization study of the association of serum uric acid and 25-hydroxyvitamin D. **(A)** Data sources for investigating the causal association of serum uric acid on 25-hydroxyvitamin D. **(B)** Data sources for investigating the causal association of 25-hydroxyvitamin D on serum uric acid.

This study is reported as per STROBE guidelines (Supplementary MR-STROBE checklist). **Figure 1** presents the schematic of our two-sample bidirectional MR analysis. We first analyzed the effect of SUA levels on 25(OH)D concentration, followed by 25(OH)D concentration’s effect on SUA levels. To be valid, an MR study needs to fulfill the following three assumptions. First, the genetic instrument of an exposure must be robustly related to the exposure of the trait of interest. Second, the selected genetic variations should not affect the outcome by other pathways that are independent of the pathway of the exposure. Last, the genetic variations are not associated with any confounder of SUA or 25(OH)D.

### 2.1 Dataset and selection of instruments for MR

#### 2.1.1 MR study of the effect of SUA on 25(OH)D

Summary-level GWAS data for SUA (expressed as mg/dl) variants were obtained from Global Urate Genetics Consortium (GUGC) GWAS databases (ieu-a-1055) (27, 28). The GUGC GWAS comprised 110,347 individuals of European ancestry, which contained 49 cohorts from the US, Austria, Australia, Japan, the UK, Germany, Italy, Finland, Switzerland, and other countries (28). Genetic variants were analyzed using MR, based on a significant genome-wide correlation with SUA (GWAS *p*-value at <5×10^−8^). Independent variants were defined by a linkage disequilibrium r2 cutoff of 0.001. A total of 27 single-nucleotide polymorphisms (SNPs) associated with SUA levels were selected from GUGC GWAS as instrumental variable, and they were not at linkage disequilibrium. We then extracted the above selected SNPs from the outcome GWAS. An SNP (proxy) would be searched for instead if a particular requested SNP was not present in the outcome GWAS. Of the 27 SNPs, 26 were directly matched in the summary data of SNP–outcome [25(OH)D] GWAS. The variance explained for a given SNP is calculated using the formula: R2 = 2×β^2^×MAF (1-MAF), where β and MAF denote the effect of the SNP on exposure and minor allele frequency, respectively. F-statistic = (β/SE)^2^, where SE is the standard error of the genetic effect (29). SNPs of SUA used to construct the instrumental variable for the MR analysis were summarized in **Supplementary Table S1**. All the SNPs show an F-statistic >10. The proportion of variance in SUA concentrations explained by all 27 independent SNPs was estimated at 6.2%. We then excluded two SNPs (rs17632159 and rs6830367) for being palindromic. An assumption of MR analysis is that the instrumental variables are not associated with confounders. In the relationship between SUA and 25(OH)D, body fat and BMI are most likely important confounders. We used PhenoScanner V2 to assess associations of these SNPs with body fat and BMI, and the *p*-value cutoff for body fat was 1×10^−5^ (30). We excluded three SNPs (rs2231142, rs6598541, and rs7193778) for being associated with body fat and BMI. Finally, 21 SNPs were selected as the instrumental variables.

#### 2.1.2 MR study of the effect of 25(OH)D on SUA

Summary-level GWAS data for 25(OH)D [expressed as one standard deviation (SD) increase in 25(OH)D concentration, SD = 21 nmol/L] variants were obtained from the IEU GWAS databases (ebi-a-GCST90000618) (31–33), which comprised 496,946 individuals of European ancestry. It was based on the large UK Biobank (UKB) sample (417,580) (32), SUNLIGHT consortium, and 31 cohorts from Europe, Canada, and USA (79,366) (33). Instrumental variables were selected according to the same steps. A total of 117 SNPs associated with 25(OH)D were selected from GWAS summarized data as instrumental variable, and they were not at linkage disequilibrium. Of the 117 SNPS, 89 were directly matched in the summary data of SNP–outcome (SUA) association estimates. SNPs of serum 25(OH)D used to construct the instrumental variable for the MR analysis were summarized in **Supplementary Table S2**. All the SNPs show an F-statistic>10. The proportion of variance in serum 25(OH)D concentrations explained by the SNPs was estimated at 2.7%. We then excluded 13 SNPs for being palindromic and 9 SNPs (rs1949633, rs2245133, rs727857, rs9847248, rs62007299, rs11264361, rs1128535, rs13294734, and rs4147536), which were associated with body fat and BMI. Finally, 67 SNPs were directly matched in the summary data of SNP–outcome (SUA) association estimates.

### 2.2 Statistical analyses

Four MR methods were used in two sets of two-sample MR analyses: (1) inverse-variance weighted (IVW) method assumes that all SNPs are valid instruments, a standard MR method for summary-level GWAS data, and our primary method for analysis; (2) weighted median method provides valid estimates even though up to 50% of the information was provided by invalid instrumental variables (34); (3) MR-Egger regression, whose slope represents the causal association estimate, is robust to invalid instruments against directional pleiotropy; and (4) weighted mode method estimates the causal effect of the subset with the largest number of SNPs by clustering the SNPs into subsets resting on the similarity of causal effects (35–37). MR-Egger regression relies on the instrument strength independent of direct effect (InSIDE) assumption that the instrument–exposure and instrument–outcome associations are independent (38, 39). We applied MR Steiger filtering to test the direction of causality for instrumental variables on exposure and outcome. Steiger filtering assumes that valid instrumental variables should explain more variation in the exposure than in the outcome; if an instrumental variable meets the criterion, the direction of this instrument is “TRUE” (40). The Steiger filtering results showed that the instrumental variables of both MR analysis had a TRUE direction.

### 2.3 Procedures of MR analysis

**Figure 2:**
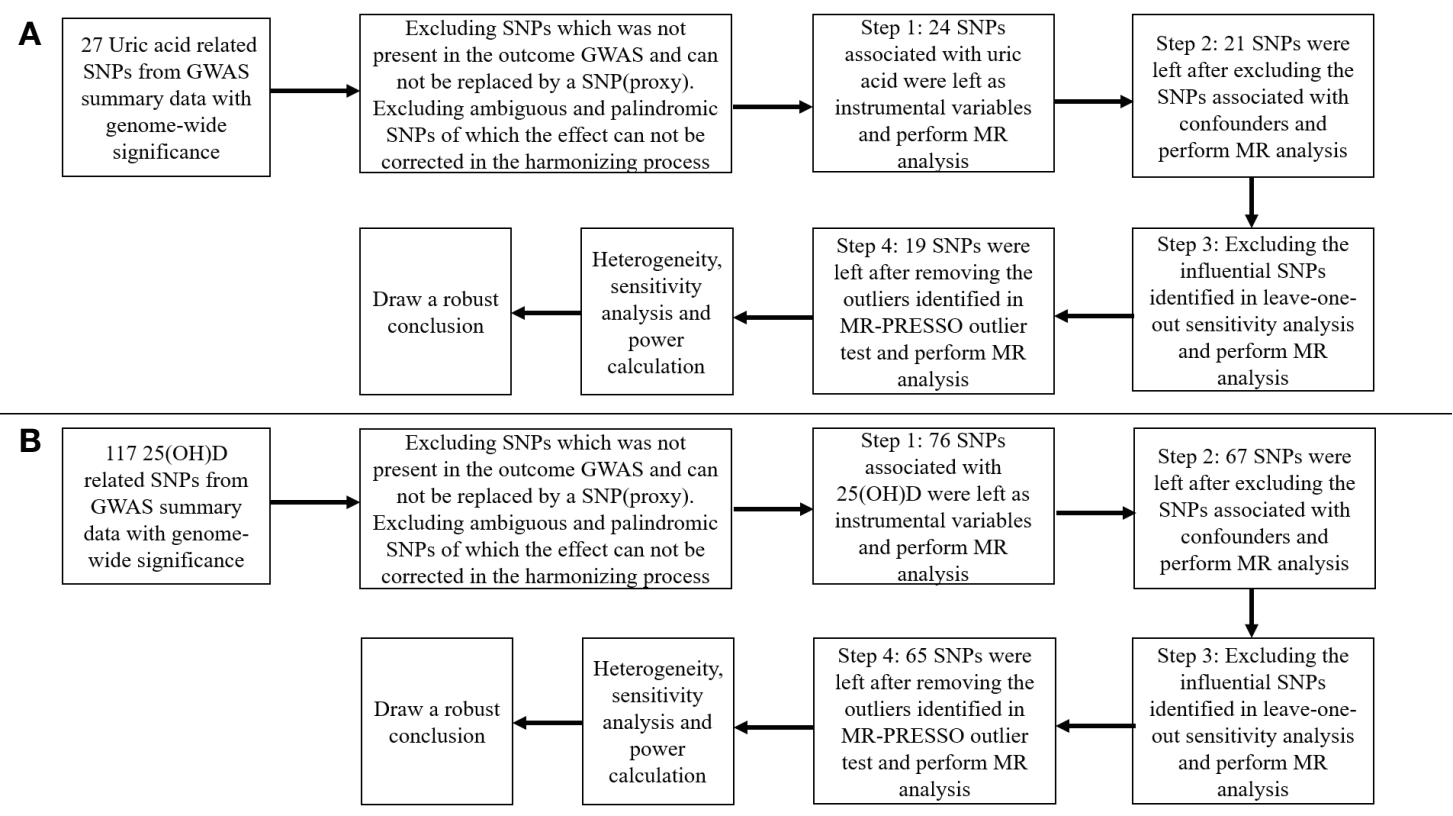
Flow chart about the analytical methods and how the MR analysis was performed step-by-step. **(A)** serum uric acid on 25-hydroxyvitamin D; **(B)** 25-hydroxyvitamin D on serum uric acid.

**Figure 2** presented the procedures of MR analysis and how the MR analysis was performed step-by-step. We first performed MR analysis with all the selected SNPs from the GWAS summary data as instrumental variables. We then performed MR analysis after excluding the SNPs associated with confounding factors. If potentially influential SNPs were identified in the “leave-one-out” sensitivity analysis, we would perform MR analysis after excluding the potential influential SNPs. At last, if the MR-PRESSO analysis proved a significant horizontal pleiotropy, we would exclude the outlier SNPs and perform MR analysis again.

### 2.4 Heterogeneity and sensitivity tests

Cochran’s Q test was used to estimate heterogeneities between SNPs. A *p*-value < 0.05 was considered statistically significant for the Cochran’s Q test. We then performed MR-Egger regression, in which the intercept represents bias due to directional pleiotropy. For MR-Egger regression, intercept *p* < 0.05 indicates SNPs with directional pleiotropy. We also used MR-PRESSO to evaluate the extent of horizontal pleiotropy. The MR-PRESSO detects outliers and removes them. MR-PRESSO consists of the MR-PRESSO global test, the MR-PRESSO outlier test, and the MRPRESSO distortion test, which relies on a regression framework with regressions based on the effect of exposure on results provided by the slope of the regression line. Outlier variants identified by the MR-PRESSO outlier test were removed step-by-step to reduce heterogeneity and the effect of horizontal pleiotropy. Leave-one-out analysis was performed to assess whether the causal estimate was driven by a single SNP. Previous MR studies used SNPs in or near genes (DHCR7, GC, CYP2R1, and CYP24A1) encoding enzymes and carrier proteins involved in vitamin D synthesis or metabolism as instrumental variables. We also performed a sensitivity analysis using 25(OH)D SNPs only in four genes with known role in vitamin D metabolism [DHCR7 (rs12785878), CYP2R1 (rs10741657), GC (rs3755967), and CYP24A1 (rs17216707)] (33).

### 2.5 Associations between exposure genetic risk score and outcome in both directions

In order to obtain the combined estimate of the relationship of exposure-influencing alleles with the outcome, MR analyses were conducted using weighted genetic risk score (GRS) as instrumental variables in both directions by using the same summary-level GWAS data described above. We conducted the analyses with the “gtx” R package (version 0.0.8), whose “grs.summary” module had the GRS function. The “grs.summary” module used single SNP association summarized data obtained from the results of the GWAS analysis, which was similar to a method that regresses an outcome onto an additive GRS. Previous studies have reported that this MR method utilizing summary-level GWAS data was equally efficient to that using individual-level data (41, 42).

All statistical analyses were conducted with R 4.1.0, and packages “TwosampleMR,” “MR-PRESSO,” and “gtx” were used in our study. A two-sided *p* < 0.05 was considered statistically significant. We used freely accessible summary-level GWAS data in this study. No original data were collected for this manuscript, and thus, no ethical committee approval was required.

## 3 Results

### 3.1 MR results of serum uric acid on 25(OH)D

**Figure 3:**
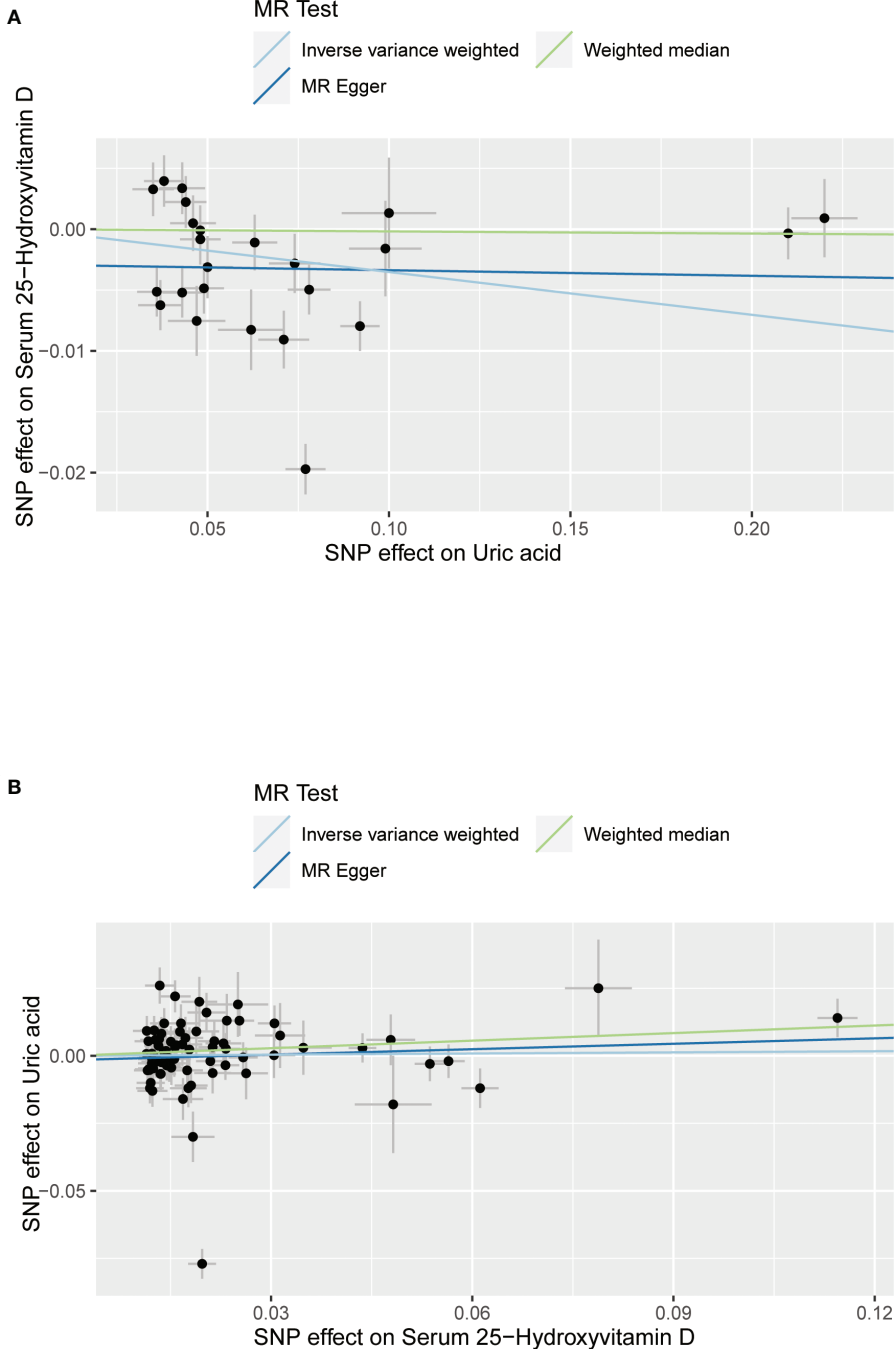
Scatter plot showing the associations of the SNP effects between serum uric acid and 25-hydroxyvitamin D. Circles indicate genetic associations between serum uric acid and 25-hydroxyvitamin D. Error bars indicate 95% confidence intervals. **(A)** Serum uric acid on 25-hydroxyvitamin D; **(B)** 25-hydroxyvitamin D on serum uric acid. MR, Mendelian Randomization; SNP, single-nucleotide polymorphism.

**Figure 4:**
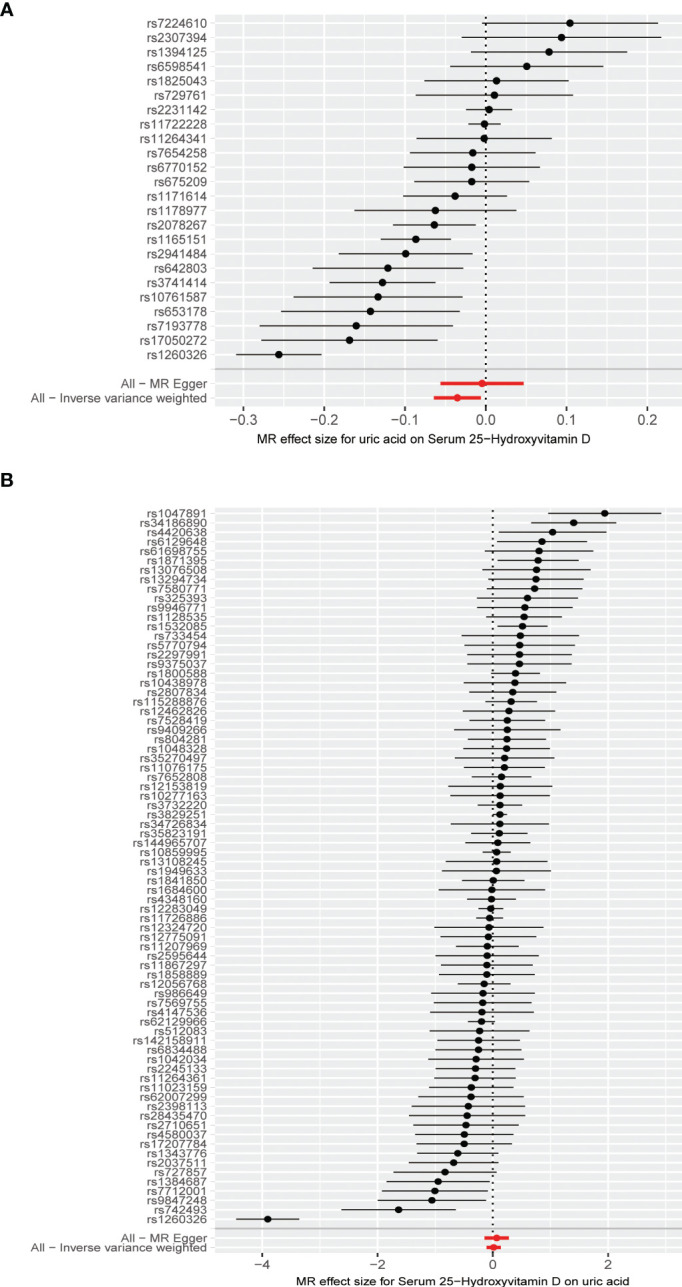
**(A)** Forest plot of causal association for uric acid on 25-hydroxyvitamin D. **(B)** Forest plot of causal association for 25-hydroxyvitamin D on uric acid.

**Figure 5:**
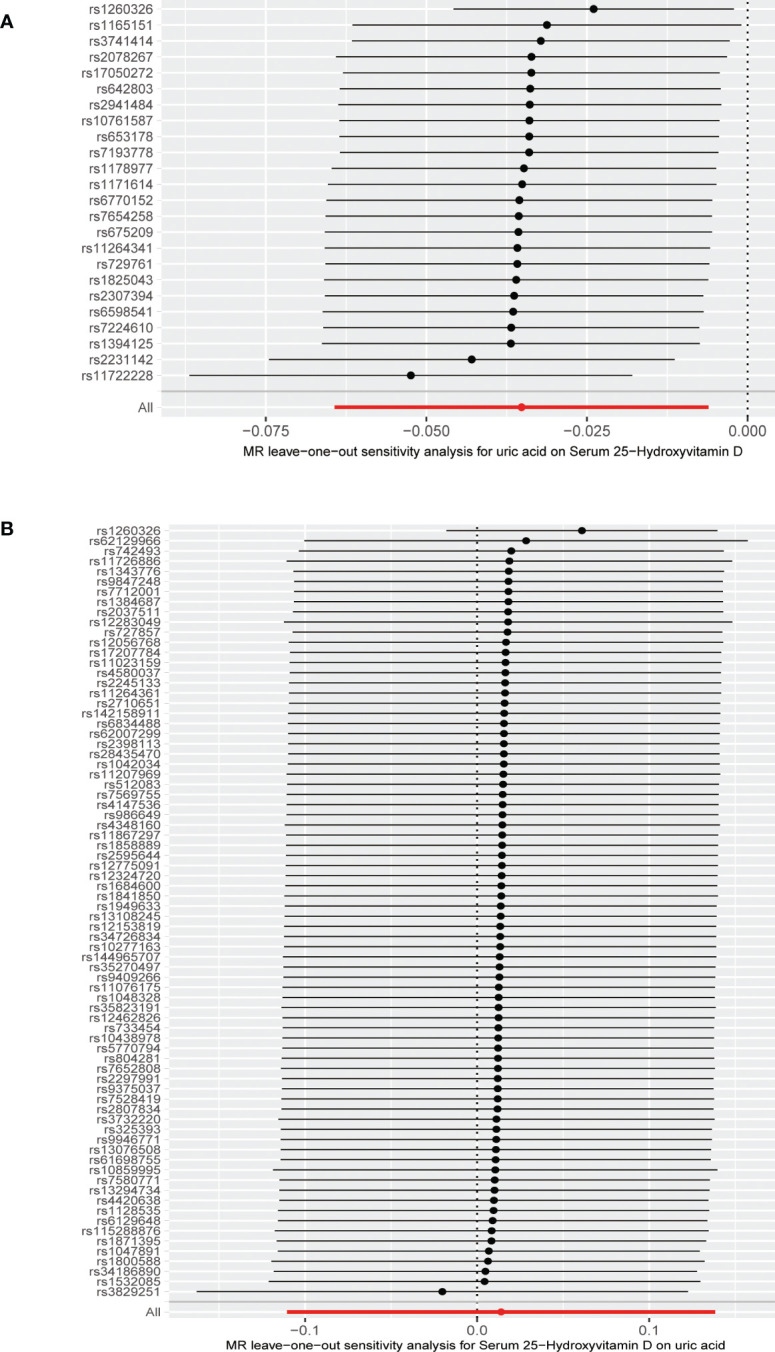
**(A)** Leave-one-out analysis of the effect of the uric acid on 25-hydroxyvitamin D. **(B)** Leave-one-out analysis of the effect of the 25-hydroxyvitamin D on uric acid.

The MR results from different methods of assessing the causal association of SUA on 25(OH)D at different steps are presented in **Table 1**. The MR results demonstrated that genetically predicted SUA was negatively related to the level of 25(OH)D in initial practice [IVW β(SE), −0.0352(0.0149); *p* = 0.0178], which suggested that a per mg/dl increase in SUA is associated with a decrease of 0.74 nmol/L of 25(OH)D. Insignificant results were found for the MR-Egger regression, weighted median, and weighted mode [MR-Egger β(SE), −0.00458 (0.0263); *p* = 0.863; weighted median β(SE), −0.00179 (0.00879); *p* = 0.838; weighted mode β(SE), −0.00622 (0.00884); *p* = 0.489, respectively] analyses. The estimated effect sizes of the SNPs on both the SUA and 25(OH)D outcomes are displayed in scatter plots (**Figure 3A**). The forest plot of MR effect size for SUA on serum 25(OH)D is presented in **Figure 4A**. After removing the SNPs which were associated with body fat and BMI, the MR results were still significant [IVW β(SE), −0.0433 (0.0167); *p* = 0.00940]. MR-Egger regression indicated that there was no notable directional pleiotropy (intercept = −0.00291, with a *p* = 0.176). The leave-one-out analysis showed that the causal association estimate was not driven by elimination of any single SNP (**Figure 5A**). The funnel plots providing an indication of where there existed directional horizontal pleiotropy for each outcome are shown in **Supplementary Figure S1A**. The funnel plot was roughly symmetrical. Cochran’s Q test showed certain heterogeneity among the SUA instrumental variable estimates based on the 24 SNPs. We then performed MR-PRESSO analysis to identify and remove outlier variants. After removing the outlier SNPs, the results in **Table 1** showed strong causal relationship between SUA and 25(OH)D [IVW β(SE), −0.0524 (0.0149); *p <*0.001; weighted median β(SE), −0.0629 (0.0150); *p <*0.001; weighted mode β(SE), −0.0602 (0.0198); *p* = 0.00718].

**Table 1:** MR estimates from different methods of assessing the causal association between SUA on 25(OH)D step by step.

	Step^#^	No. of SNP	IVW		MR-Egger		Weighted median		Weighted mode		
			β (Se)	*p*-value	β (Se)	*p*-value	β (Se)	*p*-value	β (Se)	*p*-value	
SUA and 25(OH)D.	1	24	−0.0352 (0.0149)	0.0178	−0.00458 (0.0263)	0.863	−0.00179 (0.00879)	0.838	−0.00622 (0.00884)		0.489
	2	21	−0.0433 (0.0167)	0.00940	−0.0139 (0.0319)	0.668	−0.00199 (0.0109)	0.854	−0.00799 (0.0102)		0.444
	4	19	−0.0524 (0.0149)	<0.001	−0.0887 (0.0471)	0.0770	−0.0629 (0.0150)	<0.001	−0.0602 (0.0198)		0.00718
25(OH)D and SUA	1	76	0.0139 (0.0635)	0.826	0.0671 (0.108)	0.537	0.0933 (0.0495)	0.0598	0.0562 (0.0463)		0.228
	2	67	0.0212 (0.0676)	0.754	0.0607 (0.114)	0.597	0.113 (0.0497)	0.0228	0.0523 (0.0470)		0.270
	3	66	0.0707 (0.0409)	0.0840	0.0554 (0.0688)	0.423	0.115 (0.0517)	0.0261	0.0596 (0.0498)		0.236
	4	65	0.0614 (0.0389)	0.115	0.0633 (0.0653)	0.336	0.113 (0.0494)	0.0228	0.0608 (0.0459)		0.190

Step #: 1. MR analysis with the complete selected SNPs; 2. MR analysis after removing the SNPs, which are associated with confounding factors; 3. MR analysis after excluding the influential SNPs identified in “leave-one-out” sensitivity analysis; 4. MR analysis after removing the outlier SNPs (with p-value less than threshold in MR-PRESSO outlier test). 25(OH)D, 25-hydroxyvitamin D; β, beta coefficient; Se, standard error; SNP, single nucleotide polymorphism; MR, Mendelian randomization; IVW, inverse-variance weighting.

### 3.2 MR results of 25(OH)D on serum uric acid

The MR results from different methods of assessing the causal association of 25(OH)D on SUA at different steps are presented in **Table 1**. However, no causal association of 25(OH)D on SUA was found in the initial practice [IVW β(SE), 0.0139 (0.0635); *p* = 0.826; MR-Egger β(SE), 0.0671 (0.108); *p* = 0.537; weighted median β(SE), 0.0933 (0.0495); *p* = 0.0598; weighted mode β(SE), 0.0562 (0.0463); *p* = 0.228, respectively). The estimated effect sizes of the 76 SNPs on both the 25(OH)D and SUA outcomes are displayed in scatter plots (**Figure 3B**). The forest plot of MR effect size for SUA on serum 25(OH)D are presented in **Figure 4B**. After removing the nine SNPs that are associated with confounding factors, we found 25(OH)D was positively related to the level of SUA in the weighted median method [β(SE), 0.113 (0.0497); *p* = 0.0228], while insignificant results were found for the IVW, MR-Egger, and weighted mode analyses [IVW β(SE), 0.0212 (0.0676); *p* = 0.754; MR-Egger, β(SE), 0.0607 (0.114); *p* = 0.597; weighted mode β(SE), 0.0523 (0.0470); *p* = 0.270, respectively]. The leave-one-out analysis (**Figure 5B**) showed that elimination of any SNP did not cause a change in the results. The funnel plots providing an indication of where there existed directional horizontal pleiotropy for each outcome are shown in **Supplementary Figure S1B**. However, the leave-one-out analysis, the forest plot, and funnel plots suggested that there was a potentially influential SNP (rs1260326) driving the causal link between 25(OH)D and SUA. After removing the SNP (rs1260326), no evidence of a causal association of 25(OH)D on SUA was found in IVW, MR-Egger, and weighted mode analyses [IVW β(SE), 0.0707 (0.0409); *p* = 0.0840; MR-Egger, β(SE), 0.0554 (0.0688); *p* = 0.423; weighted mode β(SE), 0.0596 (0.0498); *p* = 0.236, respectively]. The MR-Egger analysis also indicated that there was no notable directional pleiotropy (intercept = −0.0016; *p* = 0.533). Cochran’s Q test showed certain heterogeneity among the 25(OH)D IV estimates based on the 66 SNPs. We then performed MR-PRESSO analysis to identify and remove outlier variants. After removing the outlier SNPs, we still found that 25(OH)D was positively related to the level of SUA in the weighted median method [β(SE), 0.113 (0.0494); *p* = 0.0228], but a clear correlation could not be identified in the IVW, MR-Egger, and weighted mode analyses. The results of sensitivity analysis using 25(OH)D SNPs only in four genes with known role in vitamin D metabolism are presented in **Supplementary Table S3**, and no causal association between 25(OH)D and SUA was found.

### 3.3 GRS_SUA_ and 25(OH)D, GRS_25(OH)D_, and SUA

**Figure 6:**
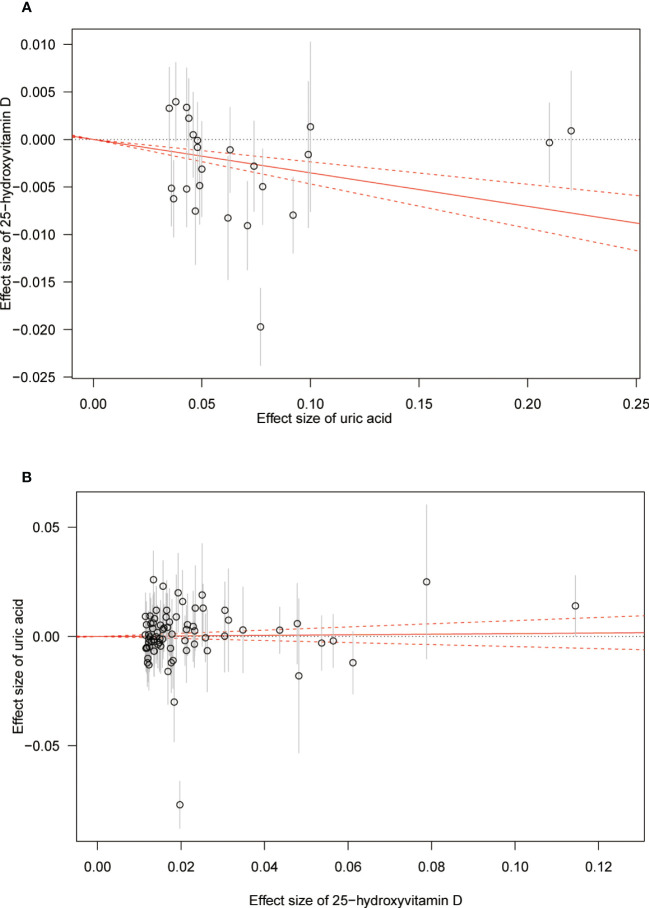
**(A)** Genetic risk score GRS_SUA_ for 25-hydroxyvitamin D. **(B)** Genetic risk score GRS_25(OH)D_ for serum uric acid. The estimate of causal association is shown by a red solid line with gradient, and 95%CIs are denoted by red dashed lines.

Consistent with the MR results of SUA on 25(OH)D, the GRS_SUA_ showed a significant effect of SUA on 25(OH)D [β (SE), −0.0351 (0.0059), *p* < 0.001] (**Table 2**, **Figure 6**). Meanwhile, consistent with the MR results of 25(OH)D to SUA. No causal effect of SUA on 25(OH)D [β (SE), 0.0132 (0.0304), *p* = 0.664] (**Table 2**, **Figure 6**).

**Table 2:** The effect of the GRS instrument of SUA on 25(OH)D and the effect of the GRS instrument of 25(OH)D on SUA.

Exposure	Outcome	Number of SNPs	β (SE)	*p-*value
SUA	25(OH)D	24	−0.0351 (0.0059)	<0.001
25(OH)D	SUA	76	0.0132 (0.0304)	0.664

GRS, genetic risk score; SUA, serum uric acid; 25(OH)D, 25-hydroxyvitamin D; SNP, single-nucleotide polymorphism; Beta, beta coefficient; SE, standard error.

### 3.4 Power of analysis

The power of our study was calculated using an online computing tool (https://shiny.cnsgenomics.com/mRnd/). We fixed the type-I error rate at 0.05 and the R2 of 0.062 and 0.027 for SUA and 25(OH)D, respectively; based on the sample sizes, our study had sufficient power (87% and 100%) to detect the causal association between SUA and 25(OH)D.

## 4 Discussion

To our knowledge, this is the first attempt that used two-sample bidirectional MR analysis to explore the causal association between SUA and 25(OH)D. Based on summary statistics form GWAS, we found a negative causal association of SUA on 25(OH)D in the IVW model. Even removing the outlier SNPs and SNPs related to confounding factors, the negative causal association was still significant. No evidence to support the causal association of 25(OH)D on SUA was found in our analysis.

The mechanisms of the relationship between Vitamin D and SUA remain unclear. Several possible explanations have been proposed. Primarily, SUA suppresses 1-α hydroxylase protein and mRNA expression in proximal tubular cells, thereby reducing the concentrations of 1,25(OH)_2_D. Febuxostat treatment could completely restore 1-α hydroxylase protein expression and partially reverse the biochemical and histological abnormalities in renal function (14). Following treatment of allopurinol, SUA level decreased and concentrations of 1,25(OH)_2_D increased significantly in chronic renal failure patients (43). SUA level was positively associated with insulin resistance and obesity. Low-serum vitamin D is common in obese people due to volumetric dilution, and insulin resistance is negatively associated with 25(OH)D concentrations (44, 45). Vitamin D deficiency induced by hyperuricemia could cause secondary hyperparathyroidism, which leads to an increase in the PTH concentration. PTH may downregulate the expression of the urate exporter ABCG2 in intestinal and renal and suppress the urate excretion, which in turn could lead to hyperuricemia (46). Previous studies found that PTH increased the incidence of hyperuricemia in a dose–response fashion (23, 47, 48). Hataikarn et al. found that vitamin D supplementation was associated with a reduction in SUA concentration after 12 weeks in participants with baseline SUA > 6 mg/dl (49).

Previous observational studies have investigated the relationship between SUA and 25(OH)D. Our previous study found that serum 25(OH)D was inversely associated with hyperuricemia in the general adult population in the US, and participants in the lowest quartile group has the highest prevalence of hyperuricemia (21). Kamil F. Faridi’s study, which aimed to investigate the associations between vitamin D and non-lipid biomarkers of cardiovascular risk, found that adults with 25(OH)D deficiency had increased odds of elevated SUA (50). Another study that involved 15,723 American adults found a lower risk of hyperuricemia in participants with higher serum 25(OH)D, dietary vitamin D, and total vitamin D intake (51). Some of the previous studies suggested that urate-lowering therapy might improve vitamin D metabolism in chronic kidney disease patients (14). To sum up, previous studies revealed a mostly negative relationship between SUA and 25(OH)D but could not determine the causal association.

Our study found a causal association of 25(OH)D on SUA using the weighted median method in steps 2–4. Four different MR methods were used in our study, and insignificant results were found for the IVW, MR-Egger regression, and weighted mode analyses. In addition, the IVW method was used as the primary analysis in our study, since the IVW method was the most powerful method to detect a causal effect (29, 35). The sensitivity analysis using 25(OH)D SNPs only in four genes with a known role in vitamin D metabolism also suggested no causal association between 25(OH)D and SUA. Thus, our study does not support a causal role of 25(OH)D on SUA.

Observational studies are prone to biases due to reverse causation and residual confounding, and our study has some advantages. We used large-scale summary-level GWAS data of SUA and 25(OH)D to perform this analysis. Our MR analysis allows us to study causal associations between modifiable exposures and risk of disease. We excluded SNPs related to confounding factors and performed bidirectional MR analysis to avoid the influence of potential confounding factors and reverse causality. No pleiotropy was found in MR-Egger regression analysis, and MR-PRESSO analysis was used to reduce the influence of horizontal pleiotropy. We performed sensitivity analyses, and the results were consistent. We also performed GRS analyses in both directions, which can support our findings from the individual SNPs. To our knowledge, this is the first study that uses two-sample bidirectional MR approach to explore the causal association between SUA and 25(OH)D.

The limitations of this study include, primarily, the following: we identified 117 SNPs to be genome-wide significant for 25(OH)D, explaining only 2.7% of the variance in 25(OH)D levels. Only 76 of the 117 SNPs remaining non-palindromic SNPs significantly associated with 25(OH)D were matched in the outcome data directly and were used as instrumental variables for 25(OH)D. Among the 76 SNPs, 26 SNPs were proxy SNPs. Using excessive proxy SNPs may produce unreliable results. In addition, the participants in our analyses are principally of European ancestry, which minimized the possibility of population stratification bias, but our results may not be representative of other ethnic groups. We used summary-level data in our MR analysis, so it was impossible to perform stratified analysis by strata of the exposure. We used datasets from different countries to reduce the overlap rate, and most of the participants were from different consortiums or cohorts. However, several cohorts were involved in both exposure and outcome GWAS datasets, and we could not get the exact overlap rate between the same cohorts; there may be a potential high overlap to cause some bias.

## 5 Conclusions

In summary, in this two-sample bidirectional MR analysis, we found a negative causal association of SUA on 25(OH)D. However, our analysis does not support a causal role of 25(OH)D on SUA. Our findings suggest that increased levels of uric acid should prompt investigation for vitamin D deficiency.

## Data availability statement

The original contributions presented in the study are included in the article/**Supplementary material**. Further inquiries can be directed to the corresponding authors.

## Author contributions

All authors helped to perform the research. Conceptualization, YH and YZ. Methodology, YH. Software, YH. Validation, YH and YZ. Formal analysis, YH. Investigation, YH. Resources, YH. Data curation, YH. Writing—original draft preparation, YH and YZ. Writing—review and editing, YZ and XZ. Visualization, YZ and XZ. Supervision, YZ and XZ. Project administration, YH, YZ, and XZ. Funding acquisition, YZ and XZ. All authors read and approved the final manuscript.

## Supplementary material

Supplementary Figure 1
**(A)** Funnel plots to visualize overall heterogeneity of MR estimates for the effect of serum uric acid on 25-hydroxyvitamin D. **(B)** Funnel plots to visualize overall heterogeneity of MR estimates for the effect of 25-hydroxyvitamin D on serum uric acid. MR, Mendelian Randomization.
