# Supplementary figures and images for: Assessment of causal associations between uric acid and 25-hydroxyvitamin D levels
Source: Front Endocrinol (Lausanne). 2022 Dec 13;13:1024675. doi: 10.3389/fendo.2022.1024675 (PMC9792848; doi:10.3389/fendo.2022.1024675)

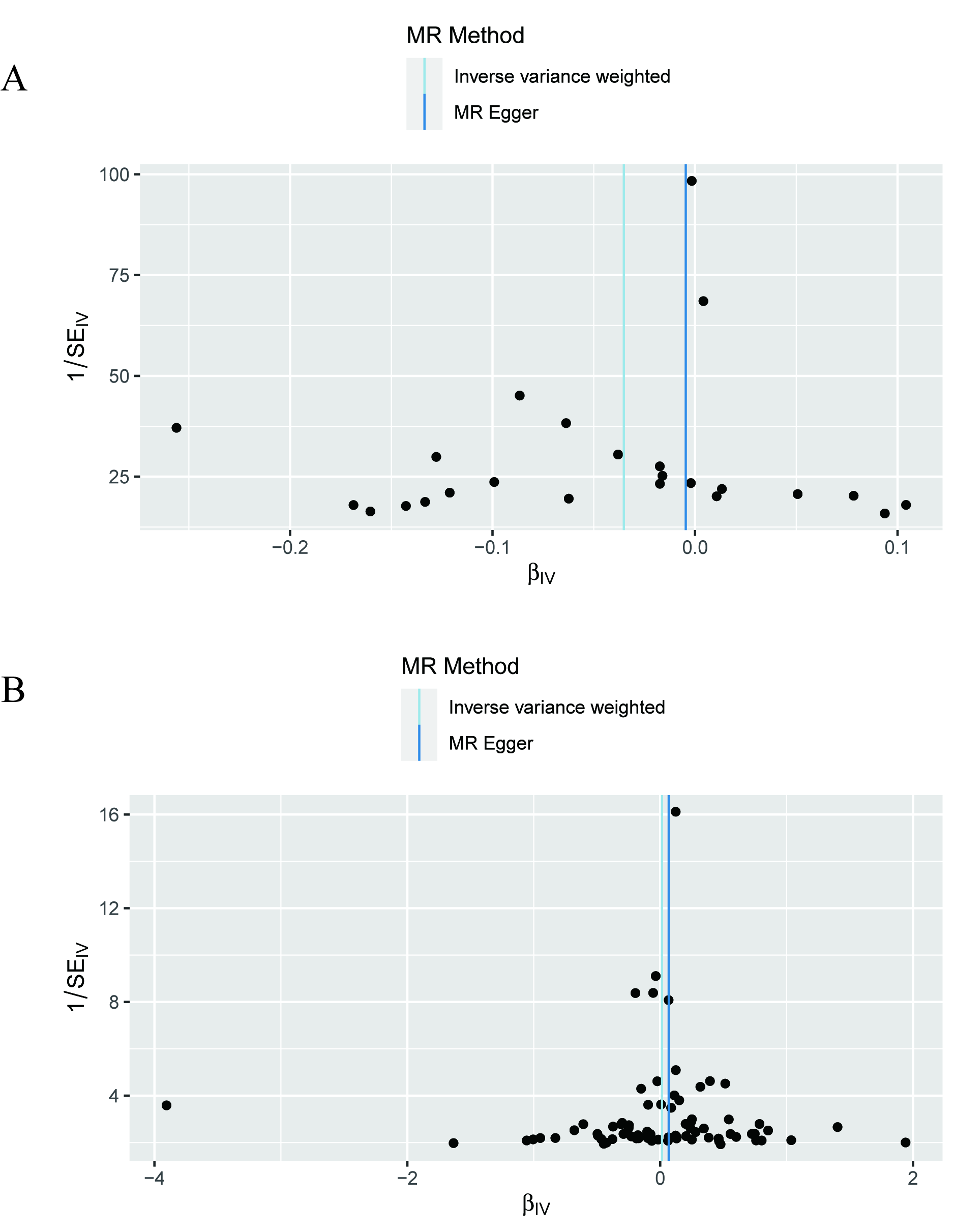

Supplement: Supplementary Figure 1 — (A) Funnel plots to visualize overall heterogeneity of MR estimates for the effect of serum uric acid on 25-hydroxyvitamin D. (B) Funnel plots to visualize overall heterogeneity of MR estimates for the effect of 25-hydroxyvitamin D on serum uric acid. MR, Mendelian Randomization. [file Image_1.tif]
