# Supplementary material for: Assessment of causal associations between uric acid and 25-hydroxyvitamin D levels
Source: Front Endocrinol (Lausanne). 2022 Dec 13;13:1024675. doi: 10.3389/fendo.2022.1024675 (PMC9792848; doi:10.3389/fendo.2022.1024675)
Supplement: Supplementary file 2 [file Table_1.docx]

Table S1. SNPs of serum uric acid used to construct the instrumental variable for the MR analysis.

| SNP | β | SE | *P*-value | R^2^ | F |
| --- | --- | --- | --- | --- | --- |
| rs10761587 | 0.0623 | 0.0091 | 9.58E-11 | 0.00078 | 46.87 |
| rs11264341 | -0.0483 | 0.0059 | 1.04E-14 | 0.001143 | 67.02 |
| rs1165151 | -0.0925 | 0.0054 | 4.52E-60 | 0.004267 | 293.42 |
| rs1171614 | -0.074 | 0.0071 | 6.48E-23 | 0.00187 | 108.63 |
| rs11722228 | 0.2057 | 0.0056 | 1.00E-200 | 0.019392 | 1349.25 |
| rs1178977 | 0.0501 | 0.0069 | 6.68E-12 | 0.000771 | 52.72 |
| rs1260326 | 0.077 | 0.0055 | 1.31E-40 | 0.002878 | 196.00 |
| rs1394125 | 0.043 | 0.0063 | 9.78E-11 | 0.000834 | 46.59 |
| rs1471633 | 0.0611 | 0.0054 | 1.40E-26 | 0.001857 | 128.03 |
| rs17050272 | 0.0372 | 0.0061 | 9.36E-09 | 0.000679 | 37.19 |
| rs17632159 | -0.0384 | 0.0061 | 2.00E-09 | 0.000628 | 39.63 |
| rs1825043 | 0.1002 | 0.013 | 6.67E-14 | 0.00102 | 63.24 |
| rs2078267 | -0.0776 | 0.0059 | 8.73E-36 | 0.003009 | 172.99 |
| rs2231142 | 0.2208 | 0.0091 | 4.44E-116 | 0.009561 | 588.73 |
| rs2307394 | -0.0352 | 0.0057 | 7.26E-09 | 0.000537 | 38.14 |
| rs2941484 | 0.0491 | 0.0055 | 3.91E-17 | 0.001189 | 79.70 |
| rs3741414 | -0.0706 | 0.007 | 9.79E-22 | 0.001798 | 101.72 |
| rs642803 | -0.0428 | 0.0054 | 4.51E-14 | 0.000909 | 62.82 |
| rs653178 | -0.0363 | 0.0054 | 2.45E-10 | 0.000659 | 45.19 |
| rs6598541 | 0.0435 | 0.0057 | 5.20E-13 | 0.000873 | 58.24 |
| rs675209 | 0.0628 | 0.0062 | 1.38E-21 | 0.001549 | 102.60 |
| rs6770152 | -0.0482 | 0.0056 | 2.66E-16 | 0.001131 | 74.08 |
| rs6830367 | 0.0512 | 0.0072 | 2.35E-11 | 0.000767 | 50.57 |
| rs7193778 | -0.0466 | 0.0079 | 2.36E-08 | 0.000522 | 34.80 |
| rs7224610 | -0.0382 | 0.0055 | 4.74E-11 | 0.000711 | 48.24 |
| rs729761 | -0.0464 | 0.0063 | 3.05E-12 | 0.000897 | 54.24 |
| rs7654258 | -0.0992 | 0.01 | 1.09E-19 | 0.001673 | 92.76 |

SNPs, single-nucleotide polymorphisms; β, beta coefficient; Se, standard error; MR, Mendelian Randomization;

Table S2. SNPs of Serum 25-Hydroxyvitamin D used to construct the instrumental variable for the MR analysis.

| SNP | β | SE | *P*-value | R^2^ | F |
| --- | --- | --- | --- | --- | --- |
| rs10277163 | -0.01433 | 0.002351 | 1.08E-09 | 0.000078 | 37.18 |
| rs1038165 | 0.011515 | 0.002056 | 2.15E-08 | 0.00006462 | 31.36 |
| rs1042034 | -0.01513 | 0.0025 | 1.45E-09 | 0.00007534 | 36.60 |
| rs10438978 | -0.01722 | 0.002644 | 7.34E-11 | 0.00008747 | 42.43 |
| rs1047891 | -0.0134 | 0.00218 | 7.96E-10 | 0.00007775 | 37.77 |
| rs1048328 | 0.03135 | 0.003744 | 5.58E-17 | 0.00014433 | 70.12 |
| rs10859995 | -0.04363 | 0.002055 | 4.60E-100 | 0.00092737 | 450.90 |
| rs11023159 | 0.048212 | 0.005725 | 3.73E-17 | 0.00014624 | 70.92 |
| rs11076175 | 0.022903 | 0.00267 | 9.64E-18 | 0.00015192 | 73.58 |
| rs111515741 | -0.04874 | 0.00779 | 3.95E-10 | 0.0000808 | 39.14 |
| rs11207969 | 0.02094 | 0.002127 | 7.14E-23 | 0.00019986 | 96.94 |
| rs11264361 | 0.017488 | 0.002341 | 7.97E-14 | 0.00011511 | 55.81 |
| rs1128535 | 0.016413 | 0.002029 | 6.06E-16 | 0.00013469 | 65.42 |
| rs115288876 | 0.078807 | 0.004982 | 2.36E-56 | 0.00051478 | 250.19 |
| rs11542462 | -0.02478 | 0.002983 | 9.72E-17 | 0.00014208 | 69.03 |
| rs11600054 | 0.068175 | 0.010148 | 1.84E-11 | 0.00009291 | 45.13 |
| rs11726886 | -0.05367 | 0.002255 | 3.08E-125 | 0.00118818 | 566.60 |
| rs117300835 | -0.33499 | 0.00886 | 1.00E-200 | 0.00294655 | 1429.48 |
| rs11791258 | 0.014081 | 0.002581 | 4.85E-08 | 0.00006129 | 29.77 |
| rs11867297 | 0.013543 | 0.002095 | 1.01E-10 | 0.00008689 | 41.81 |
| rs12056768 | -0.0232 | 0.002064 | 2.65E-29 | 0.00026143 | 126.29 |
| rs12153819 | -0.01782 | 0.003091 | 8.16E-09 | 0.00006857 | 33.24 |
| rs12283049 | -0.05646 | 0.002406 | 9.62E-122 | 0.00114409 | 550.54 |
| rs12324720 | -0.01492 | 0.002675 | 2.45E-08 | 0.00006413 | 31.10 |
| rs12462826 | -0.01321 | 0.002115 | 4.18E-10 | 0.00008134 | 39.03 |
| rs12501515 | -0.07896 | 0.002069 | 1.00E-200 | 0.00301687 | 1455.88 |
| rs1260326 | 0.019719 | 0.002074 | 1.96E-21 | 0.00018603 | 90.38 |
| rs12775091 | 0.015562 | 0.002477 | 3.33E-10 | 0.00008131 | 39.47 |
| rs13076508 | 0.025054 | 0.00451 | 2.78E-08 | 0.00006358 | 30.86 |
| rs13108245 | -0.01223 | 0.002086 | 4.63E-09 | 0.00007088 | 34.34 |
| rs1321247 | -0.02219 | 0.003366 | 4.36E-11 | 0.00008983 | 43.44 |
| rs13294734 | 0.012568 | 0.002058 | 1.02E-09 | 0.00007861 | 37.28 |
| rs1343776 | 0.018076 | 0.00245 | 1.62E-13 | 0.00011263 | 54.42 |
| rs138335 | -0.01377 | 0.002151 | 1.56E-10 | 0.00008522 | 40.95 |
| rs1384687 | -0.01687 | 0.002997 | 1.82E-08 | 0.0000653 | 31.67 |
| rs142004400 | -0.031 | 0.005596 | 3.01E-08 | 0.00006351 | 30.70 |
| rs142158911 | 0.026284 | 0.003234 | 4.43E-16 | 0.00013723 | 66.04 |
| rs144965707 | -0.03481 | 0.004218 | 1.52E-16 | 0.00014061 | 68.14 |
| rs1532085 | 0.025281 | 0.002086 | 8.60E-34 | 0.00030215 | 146.82 |
| rs1627043 | -0.04864 | 0.005661 | 8.49E-18 | 0.00015209 | 73.84 |
| rs1684600 | -0.01253 | 0.002217 | 1.59E-08 | 0.00006578 | 31.94 |
| rs17207784 | -0.01349 | 0.002171 | 5.14E-10 | 0.00007979 | 38.62 |
| rs17473257 | -0.06114 | 0.0078 | 4.59E-15 | 0.0001267 | 61.43 |
| rs1800588 | -0.0305 | 0.002469 | 4.73E-35 | 0.00031401 | 152.58 |
| rs1841850 | 0.03044 | 0.003165 | 6.73E-22 | 0.00019157 | 92.50 |
| rs1858889 | 0.013451 | 0.002031 | 3.49E-11 | 0.00009047 | 43.88 |
| rs1871395 | -0.02037 | 0.002827 | 5.72E-13 | 0.00010739 | 51.94 |
| rs1949633 | 0.011416 | 0.002086 | 4.45E-08 | 0.00006225 | 29.94 |
| rs2037511 | 0.017662 | 0.002727 | 9.41E-11 | 0.00008635 | 41.94 |
| rs2074735 | 0.029267 | 0.00412 | 1.22E-12 | 0.00010387 | 50.46 |
| rs2171427 | -0.01655 | 0.002817 | 4.26E-09 | 0.0000723 | 34.50 |
| rs2229742 | -0.02498 | 0.003314 | 4.75E-14 | 0.00011698 | 56.83 |
| rs2245133 | -0.02129 | 0.00274 | 7.80E-15 | 0.00012451 | 60.38 |
| rs2297991 | 0.012755 | 0.002256 | 1.57E-08 | 0.00006581 | 31.97 |
| rs2398113 | -0.01176 | 0.002058 | 1.10E-08 | 0.00006754 | 32.66 |
| rs2494429 | -0.01485 | 0.002673 | 2.80E-08 | 0.00006421 | 30.84 |
| rs2511279 | 0.098172 | 0.005208 | 2.98E-79 | 0.00073356 | 355.30 |
| rs2595644 | -0.01226 | 0.002097 | 4.97E-09 | 0.0000712 | 34.20 |
| rs2710651 | -0.01159 | 0.002035 | 1.23E-08 | 0.00006697 | 32.44 |
| rs2756119 | 0.012143 | 0.00211 | 8.71E-09 | 0.00006958 | 33.11 |
| rs2807834 | -0.01506 | 0.002187 | 5.66E-12 | 0.00009789 | 47.44 |
| rs28435470 | -0.01187 | 0.002148 | 3.29E-08 | 0.00006294 | 30.53 |
| rs2847500 | -0.02255 | 0.003087 | 2.77E-13 | 0.00010984 | 53.36 |
| rs290400 | -0.0131 | 0.002163 | 1.41E-09 | 0.00007637 | 36.65 |
| rs3114045 | -0.02217 | 0.00298 | 1.00E-13 | 0.00011401 | 55.36 |
| rs325393 | -0.01365 | 0.002277 | 2.03E-09 | 0.00007483 | 35.94 |
| rs34186890 | -0.01569 | 0.002318 | 1.33E-11 | 0.00009459 | 45.77 |
| rs34726834 | 0.014013 | 0.002349 | 2.42E-09 | 0.00007407 | 35.60 |
| rs35270497 | 0.015672 | 0.002682 | 5.08E-09 | 0.00007132 | 34.16 |
| rs35823191 | -0.02326 | 0.002141 | 1.65E-27 | 0.00024359 | 118.10 |
| rs3732220 | -0.04784 | 0.003633 | 1.31E-39 | 0.00035702 | 173.44 |
| rs3829251 | -0.11445 | 0.002981 | 1.00E-200 | 0.00302636 | 1474.55 |
| rs4147536 | -0.0148 | 0.00249 | 2.76E-09 | 0.00007307 | 35.34 |
| rs4348160 | -0.02584 | 0.002163 | 6.62E-33 | 0.00029387 | 142.77 |
| rs4364259 | 0.017241 | 0.002567 | 1.86E-11 | 0.00009464 | 45.12 |
| rs4420638 | -0.0193 | 0.002659 | 3.95E-13 | 0.00010841 | 52.67 |
| rs4580037 | -0.01356 | 0.002251 | 1.68E-09 | 0.00007506 | 36.31 |
| rs512083 | 0.012217 | 0.002043 | 2.23E-09 | 0.00007421 | 35.77 |
| rs57601828 | 0.011542 | 0.002082 | 2.96E-08 | 0.00006364 | 30.74 |
| rs5770794 | -0.01331 | 0.002211 | 1.74E-09 | 0.00007641 | 36.25 |
| rs6129648 | 0.014063 | 0.002106 | 2.44E-11 | 0.00009317 | 44.58 |
| rs61698755 | -0.01147 | 0.002051 | 2.25E-08 | 0.00006478 | 31.26 |
| rs61747728 | 0.030306 | 0.005269 | 8.83E-09 | 0.0000681 | 33.08 |
| rs61813875 | 0.082129 | 0.006589 | 1.16E-35 | 0.00032653 | 155.38 |
| rs61887421 | -0.03673 | 0.005978 | 8.05E-10 | 0.00007872 | 37.75 |
| rs62007299 | -0.01242 | 0.002244 | 3.12E-08 | 0.00006314 | 30.63 |
| rs62129966 | 0.061164 | 0.002764 | 1.60E-108 | 0.00100992 | 489.77 |
| rs635634 | -0.01505 | 0.002604 | 7.55E-09 | 0.00006873 | 33.39 |
| rs6438900 | 0.015049 | 0.00234 | 1.27E-10 | 0.00008627 | 41.35 |
| rs6672758 | 0.016248 | 0.002555 | 2.04E-10 | 0.0000844 | 40.43 |
| rs6834488 | -0.01445 | 0.002059 | 2.26E-12 | 0.00010191 | 49.25 |
| rs71599974 | 0.025738 | 0.002862 | 2.39E-19 | 0.00016711 | 80.88 |
| rs727857 | -0.01206 | 0.002099 | 9.27E-09 | 0.00006903 | 32.99 |
| rs733454 | 0.018855 | 0.0034 | 2.93E-08 | 0.00006351 | 30.75 |
| rs73413596 | 0.022347 | 0.003889 | 9.15E-09 | 0.00006847 | 33.01 |
| rs742493 | 0.018353 | 0.003206 | 1.04E-08 | 0.00006745 | 32.77 |
| rs7528419 | 0.021539 | 0.002432 | 8.17E-19 | 0.00016149 | 78.46 |
| rs7569755 | 0.01364 | 0.002256 | 1.49E-09 | 0.00007639 | 36.54 |
| rs7580771 | -0.01656 | 0.002665 | 5.15E-10 | 0.00007956 | 38.62 |
| rs7652808 | -0.02129 | 0.002126 | 1.36E-23 | 0.00020629 | 100.23 |
| rs7712001 | 0.011939 | 0.002062 | 7.05E-09 | 0.00007025 | 33.52 |
| rs77532868 | 0.025956 | 0.004563 | 1.28E-08 | 0.00006661 | 32.36 |
| rs7784802 | 0.01332 | 0.002116 | 3.06E-10 | 0.00008165 | 39.64 |
| rs77924615 | -0.01525 | 0.00259 | 3.94E-09 | 0.00007281 | 34.65 |
| rs77960347 | -0.05257 | 0.00906 | 6.53E-09 | 0.00006931 | 33.67 |
| rs78649910 | -0.01914 | 0.003322 | 8.33E-09 | 0.00006926 | 33.20 |
| rs7955128 | 0.013062 | 0.002039 | 1.48E-10 | 0.00008516 | 41.05 |
| rs8018720 | -0.0345 | 0.002661 | 1.94E-38 | 0.00034591 | 168.09 |
| rs804281 | 0.015895 | 0.00206 | 1.20E-14 | 0.0001228 | 59.54 |
| rs8107974 | 0.035567 | 0.003823 | 1.36E-20 | 0.00017832 | 86.55 |
| rs8121940 | -0.04356 | 0.002549 | 1.77E-65 | 0.00060178 | 292.06 |
| rs9375037 | 0.011706 | 0.002054 | 1.21E-08 | 0.00006763 | 32.47 |
| rs9409266 | -0.01678 | 0.002947 | 1.24E-08 | 0.00006687 | 32.42 |
| rs964184 | 0.040685 | 0.002989 | 3.50E-42 | 0.00038116 | 185.23 |
| rs9847248 | -0.01231 | 0.002245 | 4.19E-08 | 0.000062 | 30.06 |
| rs986649 | 0.012864 | 0.002178 | 3.51E-09 | 0.00007221 | 34.88 |
| rs9946771 | -0.0234 | 0.004077 | 9.47E-09 | 0.00006783 | 32.95 |

SNPs, single-nucleotide polymorphisms; β, beta coefficient; Se, standard error; MR, Mendelian Randomization;

Table S3 Causal association of Vitamin D on UA using MR analyses.

|  | No. of SNP | IVW |  | MR-Egger | | Weighted median | |
| --- | --- | --- | --- | --- | --- | --- | --- |
|  |  | β (Se) | *P* value | β (Se) | *P* value | β (Se) | *P* value |
| 25(OH)D and SUA | 4 | 0.0868 (0.0609) | 0.154 | -0.0976 (0.120) | 0.503 | 0.0779  (0.061) | 0.204 |

Sensitivity analysis using 25OHD SNPs only in 4 genes with known role in vitamin D metabolism (DHCR7 (rs12785878), CYP2R1 (rs10741657), GC (rs3755967), and CYP24A1 (rs17216707)).

25(OH)D, 25-hydroxyvitamin D; β, beta coefficient; Se, standard error; SNP, single nucleotide polymorphism; MR, Mendelian randomization; IVW: inverse variance weighting.
